# Meningeal Solitary Fibrous Tumor: A Single-Center Retrospective Cohort Study

**DOI:** 10.1155/2024/8846018

**Published:** 2024-01-17

**Authors:** Siyer Roohani, Yasemin Alberti, Maximilian Mirwald, Felix Ehret, Carmen Stromberger, Soleiman Fabris Roohani, Katja Bender, Anne Flörcken, Sven Märdian, Daniel Zips, David Kaul

**Affiliations:** ^1^Charité−Universitätsmedizin Berlin, Corporate Member of Freie Universität Berlin and Humboldt-Universität zu Berlin, Department of Radiation Oncology, Augustenburger Platz 1, Berlin 13353, Germany; ^2^Berlin Institute of Health at Charité−Universitätsmedizin Berlin, BIH Biomedical Innovation Academy, BIH Charité Junior Clinician Scientist Program, Charitéplatz 1, Berlin 10117, Germany; ^3^Charité−Universitätsmedizin Berlin, German Cancer Consortium (DKTK), Partner Site Berlin, and German Cancer Research Center (DKFZ), Berlin, Germany; ^4^Department of Radiotherapy, West German Cancer Center, University of Duisburg-Essen, University Hospital Essen, Essen, Germany; ^5^Vivantes Klinikum Neukölln, Department of Radiooncology and Radiotherapy, Berlin, Germany; ^6^Charité−Universitätsmedizin Berlin, Corporate Member of Freie Universität Berlin and Humboldt Universität zu Berlin, Department of Hematology, Oncology and Tumor Immunology, Augustenburger Platz 1, Berlin 13353, Germany; ^7^Charité−Universitätsmedizin Berlin, Corporate Member of Freie Universität Berlin and Humboldt-Universität zu Berlin, Center for Musculoskeletal Surgery, Campus Virchow-Klinikum, Augustenburger Platz 1, Berlin 13353, Germany

## Abstract

**Background:**

Meningeal solitary fibrous tumors (SFTs) are rare, malignant, mesenchymal tumors of the central nervous system. While surgical gross total resection is widely accepted as a positive prognostic factor for local control (LC), the role of postoperative radiotherapy (PORT) remains controversial. We sought to report our institutional experience with a particular focus on outcomes after PORT.

**Materials and Methods:**

In this single-center, retrospective cohort study, 20 patients with the primary diagnosis of histopathologically confirmed meningeal SFT were analyzed. Data on patient characteristics, imaging, treatment modalities, histopathology, and oncological outcomes were collected. LC and overall survival (OS) were assessed using the Kaplan–Meier estimator.

**Results:**

The median follow-up time was 95.8 months. After surgery only, 9 out of 11 patients (81.8%) developed a local recurrence while, after surgery and PORT, 3 out of 9 patients (33.33%) showed local failure. The 5- and 10-year LC rates were 50.5% and 40.4% in the surgery-only group and 80% at both time points in the surgery with the PORT group. In the surgery-only group, 4 out of 11 patients (36.4%) died, and 4 out of 9 patients (44.4%) died in the surgery and PORT group. OS rates after 5 and 10 years were 88.9% and 66.7% in the surgery-only group and 88.9% and 76.2% in the surgery with PORT group.

**Conclusions:**

Our findings suggest that PORT may improve LC in patients with meningeal SFT. The low incidence of meningeal SFT impedes prospective studies and requires further international collaborative efforts to exploit retrospective datasets and molecular analysis to improve patient outcomes.

## 1. Introduction

Meningeal solitary fibrous tumors (SFTs) are rare malignant mesenchymal central nervous system tumors arising from the dura of the meninges with an age-adjusted incidence of 3.77 per 10,000,000 per year [1]. Typically, 50–60-year-old patients present with a slowly enlarging mass causing symptoms due to local compression [2]. Formerly considered two separate tumor entities, SFTs and hemangiopericytoma, were found to have the same unifying NAB2-STAT6 fusion gene alteration [3]. Therefore, both entities were combined and classified as SFT/hemangiopericytoma in the World Health Organization (WHO) classification of central nervous system tumors in 2016 [3, 4]. Eventually, the term hemangiopericytoma was abandoned and replaced with SFT in the WHO classification of central nervous tumors in 2021 [5]. The WHO classification stratifies SFTs into three grades based on mitotic activity and necrosis, both correlating with the prognosis [6, 7].

Multiple large, retrospective studies have found gross total resection (GTR) to be the most important factor for local control (LC) [1, 8–11]. The value of postoperative radiotherapy (PORT) remains controversial [8]. Although no prospective studies exist due to the rarity of meningeal SFTs, multi-institutional retrospective studies suggest a benefit of PORT for LC without translating to improvements in overall survival (OS) [9, 12]. Moreover, WHO grade 3 histology was shown to be a significant negative prognostic factor for the 10-year distant metastasis-free survival [9]. This study analyzed patients with meningeal SFT treated with surgery only or surgery with PORT and their impact on oncological outcomes.

## 2. Materials and Methods

In this retrospective, single-center retrospective cohort study, we included 20 adult patients with the primary diagnosis of a histopathologically confirmed and localized meningeal SFT treated with surgery alone or surgery and PORT between 1982 and 2021. We excluded patients below 18 years of age and SFTs of other locations. We reviewed data on the patient characteristics, imaging, pathology, surgical, and radiotherapy (RT) treatment characteristics and outcomes. LC was defined as the time of unchanged or decreased SFT volume after therapy on follow-up imaging with cranial magnetic resonance imaging assessed by a board-certified neuroradiologist. Patients not developing a local recurrence within the observed timeframe were censored on the date of the last available radiographic follow-up. OS was defined as the time from primary diagnosis to death by any cause, with patients being censored on the last available follow-up. Data on survival status were obtained from the municipal registry. Ranges, medians, standard deviations, and means for continuous variables were used. LC and OS were assessed using the Kaplan–Meier estimator. Statistical analysis was performed with GraphPad Prism v.9.3.1 (GraphPad Software, San Diego, CA, USA). The study was approved by the institutional review board (EA1/072/23).

## 3. Results

Patient, tumor, and treatment characteristics are summarized in Table 1. We included 20 patients with a median clinical follow-up of the entire cohort of 95.8 months. The median age at diagnosis was 45.5 years, and 50% of patients were males. Nine patients were treated with surgery and PORT while 11 patients received surgery alone. Nine patients were treated between 1982 and 2005; eleven patients were treated between 2006 and 2021. Age and sex distributions were similar between the surgery-only and surgery and PORT group. While half of the entire cohort had grade 3 (high-grade) histology, the distribution was comparable between both groups. On average, tumors were larger in the surgery + PORT group with a median tumor size of 5.1 cm compared to 4.0 cm in the surgery-only group.

**Figure 1:**
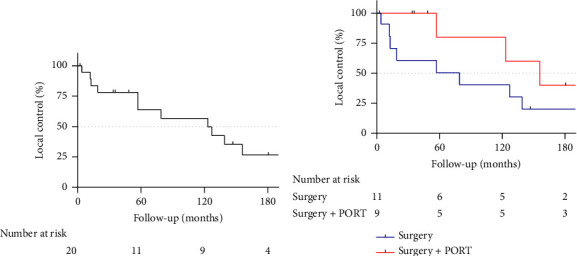
(a) Local control in the entire study cohort. (b) Local control between surgery alone (surgery) and surgery with postoperative radiotherapy (surgery + PORT).

Oncological outcomes are summarized in Table 2. Median follow-up times were similar between both groups, with 94.9 months in the surgery-only group and 96.7 months in the surgery + PORT group. About one-third of all patients experienced local disease recurrence within 5 years (5-year local control rate of 63.8%, Figure 1(a)). After surgery only, 9 out of 11 patients (81.8%) developed a local recurrence while, after surgery and PORT, 3 out of 9 patients (33.33%) developed a local recurrence. With the addition of PORT, the LC curves display diverging trends in the first 5 years after therapy completion (Figure 1(b)). The 5- and 10-year LC rates were 50.5% and 40.4% with surgery only and 80% at both time points with the addition of PORT to surgery.

**Figure 2:**
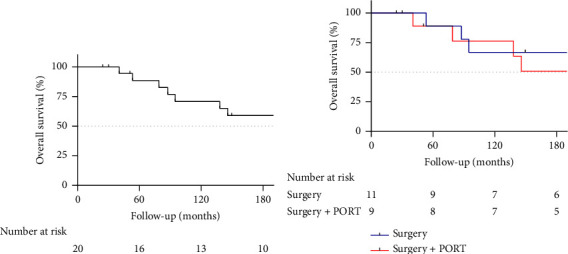
(a) Overall survival in the entire study cohort. (b) Overall survival between surgery alone (surgery) and surgery with postoperative radiotherapy (surgery + PORT).

The survival rates in the entire SFT cohort were 5- and 10-year OS of 88.5% and 70.8%, respectively (Figure 2(a)). In the surgery-only group, 4 out of 11 patients (36.4%) died, and 4 out of 9 patients (44.4%) died in the surgery and PORT group. OS rates after 5 and 10 years were 88.9% and 66.7% in the surgery-only group and 88.9% and 76.2% in the surgery with the PORT group (Figure 2(b)). Three patients developed distant pulmonary and abdominal metastases more than 5 years after receiving surgery alone. No distant pulmonary metastases were seen in the surgery with the PORT group.

## 4. Discussion

Herein, we report our single-institutional retrospective cohort study on 20 patients with localized meningeal SFT. The addition of PORT to surgery displayed a favorable trend suggesting a possible clinical benefit on LC. The diverging trends in LC rates (5-year LC rates of 63.5% for surgery only vs. 80% for surgery and PORT, respectively) may solidify with larger sample sizes. The findings did not translate into improved OS.

Our findings confirm previous larger multicenter retrospective studies on meningeal SFT. In a cohort of 48 patients from seven international high-volume sarcoma centers, PORT suggested beneficial effects on LC (5-year LC 60% in surgery only vs. 90% in surgery + PORT, respectively, *p* = 0.052) close to reaching statistical significance without a benefit on OS [12]. Compared to the present study, the authors analyzed a larger, multi-institutional cohort with similar follow-up periods and detected comparable improving LC trends without substantial impacts on OS [12]. Similarly, a single-institutional analysis of 39 patients showed significant improvements in the combined endpoint local recurrence-free survival by the addition of PORT after GTR and STR [13]. A more recent multi-institutional retrospective study by Lee et al. comparing 85 meningeal SFT patients with surgery + PORT to 48 patients with surgery only revealed a clear benefit of PORT on LC which was sustained in the multivariable Cox regression analysis [9]. Importantly, the positive effect of PORT on LC was present after both, GTR and STR, when compared to GTR and STR alone [9]. The authors did not find a beneficial effect of PORT on OS [9]. In contrast to that, a previous study on 52 patients by the group of Lee et al. did find a benefit in OS by adding PORT to surgical excision [14]. The pattern of recurrence analysis in both studies revealed that PORT improved local tumor recurrences while the proportion of regional or distant metastases increased, suggesting that PORT as a local oncological treatment modality improves LC while having less preventive effects on the risk of developing distant metastases [9, 14]. Moreover, all patients with metastases had well-controlled primary tumors and only patients with metastatic disease died [14]. This may imply that PORT has detrimental effects on survival. However, PORT prolonged the time to both, local recurrence and metastatic recurrence [14]. The authors, therefore, conclude that the benefits of PORT on OS stem from the delay in local and distant disease recurrence and the latter being the most common cause of death in the investigated cohorts [9, 14].

A recently published and comprehensive meta-analysis of 27 studies including 1,348 patients with meningeal SFT has revealed significant improvements by the addition of PORT on the combined endpoint progression-free survival (PFS) [8]. Interestingly, and in contrast to a number of previous studies and our findings, the meta-analysis did find improvements in 3- and 5-year OS by the addition of PORT; however, the benefit was not evident in the 10-year analysis [8]. Another main result of the meta-analysis is the importance of the extent of resection for the PFS. Significant improvements in 1-, 3-, 5-, and 10-year PFS were evident when GTR was achieved in comparison to STR [8]. The essential role of the extent of resection for LC was confirmed in multiple other studies [10, 15, 16]. In our cohort, the surgery + PORT group also had a larger proportion of GTR; however, the sample sizes and observed differences are not sufficient to draw firm conclusions. In the present study, three patients developed a distant recurrence with pulmonary and abdominal metastases more than five years after initial surgical therapy. Two of these patients were classified as grade 3 according to the WHO classification. Similar patterns were evident in previous studies, where WHO grade 3 was a significant negative prognostic factor for 10-year distant metastasis-free survival and OS [9, 17, 18]. This finding confirms previous studies and stresses the importance of long-term follow-up for patients with meningeal SFT, as distant extracranial recurrences can occur up to several years after therapy [7, 19, 20].

The low incidence of meningeal SFT impedes large prospective clinical trials [1]. In an attempt to overcome this epidemiological challenge and to improve the classification of SFTs in different locations, Bieg et al. applied unsupervised next-generation sequencing-based gene expression profiling on 44 SFT samples, among which 14 were meningeal SFTs [21]. The analyzed gene expression profiles were tested in a validation cohort of 29 SFT samples. The authors found distinct molecular profiles depending on the anatomical location of the SFT, thereby improving the characterization and establishing a potential tumor biological stratification method for clinical outcome differences in SFT patients [21]. Intriguingly, the analyses were retrospectively conducted with common formalin-fixed, paraffin-embedded samples making the method attractive for broader clinical applications and molecular-targeted tumor therapies. A similar molecular biological approach to characterization of SFTs was taken by Chelsky et al. who recently presented preliminary data on a methylome-based tumor profiling for SFTs of all locations [22]. The authors analyzed the methylation patterns of 28 SFT samples using a methylation-based tumor classifier by the German Cancer Research Network and validated results using publicly available samples [23]. The analysis revealed three distinct methylation-based groups of SFTs. Importantly, the intracranial SFTs were all correctly classified. Although the preliminary data did not yet find a difference in risk stratification among the three groups, the analysis highlighted the heterogeneity of SFTs and will potentially increase its discriminative power as more samples are added to the classifier [22, 24].

## 5. Limitations

The present study is limited by the small patient population, retrospective nature, and single-institutional data. Moreover, patients receiving surgery and PORT were younger and had larger tumors than patients receiving surgery alone, indicating that the treatment decision-making was inherently affected by the patient's baseline characteristics. Our study supports previous data that adding PORT to surgical resection may improve local tumor control. Future international effort is required to overcome epidemiological challenges, exploit retrospective datasets, and apply molecular analyses to further characterize this rare tumor entity and improve outcomes for patients.

## Figures and Tables

**Table 1:** Patient, tumor, and treatment characteristics.

	Total (*n* = 20)	Surgery (*n* = 11)	Surgery + PORT (*n* = 9)
Median age, years (range)	45.5 (24–74)	53.0 (34–74)	45.0 (24–54)
Median follow-up, months (IQR)	95.8 (138.7)	94.9 (138.9)	96.7 (131.9)
Sex (% female: % male)	50 : 50	45.4 : 54.6	55.6 : 44.4
WHO grade (%)			
Grade 1	10	18.2	0
Grade 2	35	27.3	44.4
Grade 3	50	54.5	44.4
N/A	5	0	11.1
Resection status			
GTR	50	45.5	55.6
STR	10	9.1	11.1
Biopsy	5	0	11.1
N/A	35	45.5	22.2
Median maximum tumor diameter, cm (SD)	4.6 (2.0)	4.0 (1.7)	5.1 (2.5)

Cm: centimeter; GTR: gross total resection; IQR: interquartile range; N/A: not available; PORT: postoperative radiotherapy; SD: standard deviation; STR: subtotal resection; WHO: World Health Organization.

**Table 2:** Oncological outcomes.

	Total (*n* = 20)	Surgery (*n* = 11)	Surgery + PORT (*n* = 9)
Median follow-up (months)	95.8	94.9	96.7
5-year local control (%)	63.8	50.5	80
5-year overall survival (%)	88.5	88.9	88.9
10-year local control (%)	56.7	40.4	80
10-year overall survival (%)	70.8	66.7	76.2
Distant metastases (%)	15	27.3	0
Time to distant metastases (months, median)	173.1	173.1	—

PORT: postoperative radiotherapy.

## Data Availability

Data are available upon request from the corresponding author.

## Abbreviations

GTR: Gross total resection
LC: Local control
OS: Overall survival
PFS: Progression-free survival
PORT: Postoperative radiotherapy
RT: Radiotherapy
SFT: Solitary fibrous tumor
STR: Subtotal resection
WHO: World Health Organization.
